# Insights on monkeypox disease and its recent outbreak with evidence of nonsynonymous missense mutation

**DOI:** 10.2144/fsoa-2023-0048

**Published:** 2023-06-24

**Authors:** Shaza M Elhusseiny, Abraam S Bebawy, Bishoy T Saad, Khaled M Aboshanab

**Affiliations:** 1 Department of Microbiology & Immunology, Faculty of Pharmacy, Ahram Canadian University (ACU), 4th Industrial Area, 6th of October City, Cairo, 12566, Egypt; 2 Department of Genomics, HITS Solutions Co., Cairo, 11765, Egypt; 3 Department of Bioinformatics, HITS Solutions Co., Cairo, 11765, Egypt; 4 Department of Microbiology & Immunology, Faculty of Pharmacy, Ain Shams University, Organization of African Unity St., Cairo, Abbassia, 11566, Egypt

**Keywords:** anti-virals, epidemiology, monkeypox virus, mutation, *orthopoxviruses*, transmission

## Abstract

The 2022 monkeypox outbreak has created a new global health threat and pandemic. Monkeypox virus is a descendant of the genus *Orthopoxvirus*, producing a febrile skin rash disease in humans. Monkeypox is zoonotic transmitted and transmitted from human to human in several ways. Even though this disease is self-limited, it creates important community health worries due to its inconvenience and widespread complications. Herein, we discussed the up-to-date current situation of monkeypox regarding its epidemiology, clinical manifestations, current in-use therapeutics, necessary protective measures, and response to potential occurrences considering the recent pandemic. Also, in this review, a comparative genomic analysis of the recent circulating strains that have been recovered from various countries including, Egypt, USA, Spain, Japan and South Africa has been investigated.

## Plain language summary

The recent monkeypox outbreaks in 2022 have created a new global health threat because of their high number of cases and the speed of infection. Infection of monkeypox is characterized by a febrile rash disease in humans and characteristic skin lesions. Monkeypox is transmitted to humans via various routes including, direct contact with infected humans or animals, or vial droplet means. Although, the disease is self-limited; however, it can cause important public health consequences, particularly in pediatric, immunocompromised individuals and during pregnancy. This review introduces the monkeypox illness epidemiology, clinical manifestations, genomic mutation of the circulating viral strains, and management.

Viruses are still responsible for large numbers of new cases of medically significant recurrent infections and widespread sets of various and serious pathological conditions affecting both humans and animals [1]. Viruses are the main source of some of the most dreaded and dreadful human illnesses because of their ability to disseminate rapidly causing high morbidity and mortality worldwide [2]. Additionally, many viruses have dual uses as bioweapons and devices of mass destruction [3]. Repeated outbreaks of potentially endemic zoonotic diseases in various locations in Africa, such as ebola, Rift Valley fever, chikungunya fever, and dengue fever, cause significant community health problems to local, mainland, and worldwide health security and continue to pose a threat [4,5]. In Nigeria, 40 years after the eradication of smallpox, the monkeypox virus recently caused varicella zoster and a severe smallpox-like rash syndrome [6]. The monkeypox 2022 outbreak poses a new worldwide health danger [7]. About 3413 confirmed cases and several deaths from 50 countries have been reported by the WHO in the period between 1 January and 22 June 2022 was reported [8]. By July 8, the cumulative number of confirmed cases had risen to 9069 [8]. Due to the rapid growth in monkeypox cases, on 23 July 2022, the WHO announced the escalation of the international monkeypox epidemic as a public health crisis of global interest. Monkeypox was first discovered in the monkey protectorate in 1958 and was later discovered in 1970 on a 9-month-old child in the Democratic Republic of the Congo [9]. Monkeypox is clearly unusual in non-endemic zones and these uncommon incidents are becoming progressively predominant [9]. Monkeypox is usually not widely spread as it needs direct contact with the infected cases. Furthermore, environmental influences, human hygiene, socioeconomic, healthcare, microbial adaptation, demographic phenomena, and public health policies are critical factors in terms of viral spread and incidence of the viral outbreak and therefore have to be greatly considered. Therefore, as the recent outbreak of monkeypox is unusual and dissimilar from previous ones, a complete thoughtful of monkeypox, including contributing pathogens, various and probable modes of transmission, viral host interaction, epidemiology, possible risks, clinical appearances, diagnosis, management including, the prevention and control strategies, is still urgently required and has to be deeply investigated.

## The Monkeypox virus

Monkeypox is a descendant of the genus *Orthopoxvirus*, where four viruses, have been reported to cause human infections and these including the monkeypox virus, vaccinia virus, cowpox virus, and the eradicated variola virus [10]. *Orthopoxvirus* genus is a member of the family; Poxviridae, the large, enveloped viruses, and their genome comprises linear, double-stranded DNA [11]. The genomics of the Monkeypox virus (MPXV) has been extensively analyzed and reported, although little is known about the virus-encoded proteome. *Orthopoxviruses* have plastic genomes characterized by a central region occupied by core genes and peripheral regions with variable gene content and carrying “accessory” genes that mainly encode proteins involved in host–virus interactions [11]. Analysis of the genomes reveals that the strain is similar to isolates from West Africa and differs from the 2018–2019 isolates by 50 single-nucleotide polymorphisms [12]. Around half of the genes are fundamental for viral multiplication, and the other half is mostly involved in host-viral interactions [13]. Monkeypox virus, unlike many DNA viruses, as it is mostly replicated in the cytoplasm rather than in the nucleus because it generates proteins required for both gene expression as well as for viral replication [14]. Monkeypox strains have been classified in two main clades (West African and Central African clades). These clades correlate with the different epidemiological monkeypox outbreaks. For instance, the first two clades include the majority of isolates linked to outbreaks from the Democratic Republic of Congo (DRC) (clade 1), formerly known as the Congo Basin clade, and from West Africa (monkeypox clade 2) [15]. Clade 1 (Congo Basin clade) is more clinically severe, with higher mortality and transmission rates. However, clade 2 monkeypox isolates are accompanied by milder infection, lower mortality rates, and reduced transmissibility [16]. Moreover, a new proposal for classifying monkeypox has been established to group isolates into three clades. Interestingly, clade 3 (monkeypox clade 3) includes isolates originating from the 2017 to 2019 outbreaks [17] and genomes from the most recent 2022 outbreak diverging emerging lineages that are currently under investigation [17]. The West African and Congo Basin groups differ in terms of genetic, geographic, and phenotypic variations, with definite epidemiological and clinical variations [18]. Unlike the West African group, the Central African tribe might be connected with more serious pathological conditions, superior human-to-human spreadability, and a high mortality rate [19].

## Monkeypox epidemiology

Although smallpox which is caused by the variola virus, is the only known human viral disease without an animal reservoir, the monkeypox virus has a broad variety of authorized animal reservoirs [20]. Human monkeypox is a rare smallpox-like disease first identified in 1958 in trapped monkeys in Denmark [21]. Five major monkeypox outbreaks have been reported, occurring in 1970, 1996–97, 2003 and 2018. The most recent of them, is the still-ongoing multi-country outbreak 2022, causing more than 6000 cases in over 50 non-endemic countries on multiple continents. Human monkeypox occurs in cities where there is a great probability of physical contact with diseased animals [22]. Previously, the largest outbreak outside Western and Central Africa occurred in 2003, when 47 cases in the Midwestern USA were linked to contact with prairie dogs from a distributor that also imported rodents from Ghana [23]. Moreover, monkeypox is an endemic disease in several African countries including, Gabon, Ghana, Cote d'Ivoire, Liberia, Nigeria, the Republic of Congo, Benin, Cameroon, the Focal Republic of Africa, Sierra Leone and Sudan [24]. Nonetheless, new cases have been detected in other countries outside Africa in recent years. In 2003, a reported case of monkeypox occurred in the USA, resulting in 53 human cases [25]. Monkeypox recently attracted the attention of media, politics, and science globally following the identification in 2018 in the UK of 3 separate patients determined to have monkeypox [26]. Moreover, on 25 May 2022, a history of realized cases has been reported from non-endemic countries such as Argentina, Israel, Switzerland, Australia, Canada, United Arab Emirates, and Morocco are among the nations documenting cases [27]. Besides, cases of monkeypox have been reported in some Asian countries in Western countries [28]. Outbreaks around the world are normally connected to people who have recently come back from the endemic regions. The now largest ongoing outbreak of monkeypox began in April 2022 and is linked to more than 57,000 cases across 103 countries, predominantly in the USA, Europe, and Brazil [29]. Egypt officially detected its first monkeypox case in early September 2022 www.who.int/emergencies/disease-outbreak-news (accessed on 06 June 2023). On the other hand, the WHO unveiled a new episode in May 2022, leading to a pattern shift with over 780 confirmed cases from 27 nonendemic areas of the monkeypox virus and no history of travel to endemic regions [30].

## Genomic mutation of the circulating viral strains

**Figure 1.**
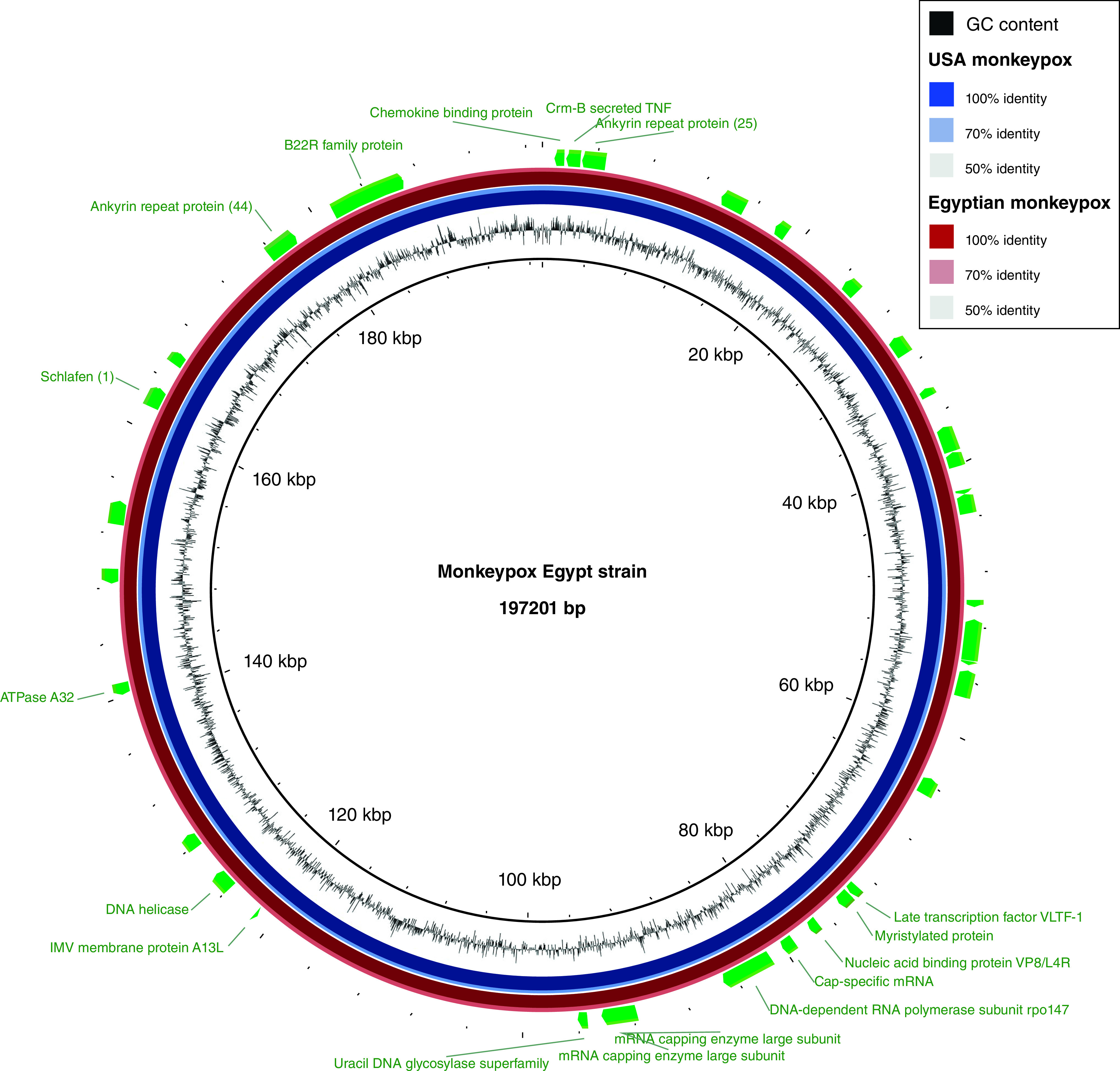
Circular genome map The monkeypox complete genome DNA sequence of the Egypt strain. The monkeypox complete genome DNA sequence of the Egypt strain “NCBI accession code, OP597769.1” (www.ncbi.nlm.nih.gov/nuccore/OP597769.1/ (accessed March 2023) was compared with homologous complete genomic DNA sequences of the USA “NCBI accession code, OP752103.1” www.ncbi.nlm.nih.gov/nuccore/OP752103.1/ (accessed March 2023). The creation of the circular image and comparison with other reported similar plasmids were performed using the BLAST Ring Image Generator (BRIG) tool v0.95 (https://sourceforge.net/projects/brig/, accessed 25 October 2022). The open reading frames (ORFs) at which nonsynonymous single nucleotide polymorphisms (SNPs) of the monkeypox strain isolated in Egypt is indicated by the green arcs (clockwise or anticlockwise according to frame direction of the detected ORFs).

The monkeypox complete genome DNA sequence of the Egypt strain “NCBI accession code, OP597769.1” (www.ncbi.nlm.nih.gov/nuccore/OP597769.1/ (accessed March 2023) was compared with homologous complete genomic DNA sequences of the USA “NCBI accession code, OP752103.1” www.ncbi.nlm.nih.gov/nuccore/OP752103.1/ (accessed March 2023), Japan “NCBI accession code, LC722946.1” www.ncbi.nlm.nih.gov/nuccore/LC722946.1 (accessed March 2023), Spain “NCBI accession code, ON838939.1” www.ncbi.nlm.nih.gov/nuccore/ON838939.1 (accessed March 2023) and South Africa “NCBI accession code, ON918611.1” www.ncbi.nlm.nih.gov/nuccore/ON918611.1 (accessed March 2023) using Multiple Alignment using Fast Fourier Transform (MAFFT software, hwww.ebi.ac.uk/Tools/msa/mafft/ (accessed March 2023). The Egyptian monkeypox isolate was coded hMpxV/Egypt/MOH-NRC-0002/2022, and was collected by the Egyptian Ministry of Health on 26 September 2022 from infected humans (*Homo Sapiens*) and the complete genome (197201 bp) was deposited and released to the public on the NCBI GenBank database on 7 October 2022. The respective multiple sequence alignments of the respective genomes were carried out to highlight the nonsynonymous single nucleotide polymorphisms (SNPs) and detect the reflected missense or non-sense SNPs of the coded proteins. We have aligned several sequences of several circulating strains for each country (USA, Spain, Japan, and South Africa) and we found that almost all the deposited sequences in the NCBI GenBank database were almost identical on the nucleotide levels (98–100% identity) with no missense or nonsense mutations could be detected. The result of this analysis and the complete list of the accompanied changes is displayed in Table 1. The results showed that the complete genome of the USA, Spain, Japan, and South Africa strains showed no SNPs when they were compared with each other. However, the SNPs were determined in 34 open reading frames (ORFs) of the complete genome of the Egyptian strain out of the 178 ORFs (19.1%) of the complete genome (Figure 1). The circular genomic map showing the ORFs on which SNPs were detected of the monkeypox complete genome DNA sequence of the Egyptian strain when compared with the USA strain is depicted in Figure 1. As presented in Table 1, the SNPs detected in each ORF were either one, two, three, or four missense SNPs, with a percentage of, 79.4%, 11.8%, 8.8%, and 2.9%, respectively. Whether these SNPs are associated with the pathogenicity, spread of infection as well as in protection efficiency of the commercially-available vaccines still has to be investigated and biochemically identified. Further investigation should be done to explore the effect of such SNPs on both the spread and the response of the virus for current therapeutics as well as for the response to current vaccines. However, this type of study needs extensive time and lab facilities as well as trained personnel to be accomplished and to explore the effect of each SNP and I think this could be done by retrospective studies to isolate the virus strain from the infected case, analyze the genome sequence, identify the SNPs and correlate them with the response to therapy of protection by vaccinations.

**Table 1.** The Genomics Mutation represented by nonsynonymous single nucleotide polymorphism (SNP) of the circulating Egyptian strain of monkeypox as compared with homologous genome sequences of the USA, Spain, Japan, and South Africa Strains circulating Strains.

Nucleotide position in Egyptian strain	Gene name	Encoded protein	Protein function	Amino acid in Egyptian	Amino acid in USA	Amino acid in Spain	Amino acid in Japan	Amino acid in South Africa
1283	OPG001	Chemokine binding protein	Competitively bind and inhibit the interactions of chemokines with cognate receptors	Leu	Ser	Ser	Ser	Ser
2612	OPG002	Crm-B (secreted TNF-alpha-receptor-like protein)	Blocks the binding of TNF to high-affinity TNFRs on the cell surface.	Ser	Phe	Phe	Phe	Phe
3839	OPG003	Ankyrin repeat protein-25	Mediate protein-protein interactions	Asp	Asn	Asn	Asn	Asn
13535,13537, 13539 & 14021	OPG025	Ankyrin repeat protein-14	Mediate protein-protein interactions	Thr, Tyr and Ala	Ile, Gln and Asp	Ile, Gln and Asp	Ile, Gln and Asp	Ile, Gln and Asp
18813	OPG031	C4L/C10L-like family protein	IL-1 receptor antagonist	Asn	Asp	Asp	Asp	Asp
25093	OPG040	Serpin	Inhibit proteases	Gln	Arg	Arg	Arg	Arg
30388, 30657 & 31074	OPG047	Kelch-like protein-2	Interact with Cullin3 to form E3 ligase complexes that mediate the ubiquitination of substrate proteins.	Gly, Asn and Arg	Ile, Asp and Cys	Ile, Asp and Cys	Ile, Asp and Cys	Ile, Asp and Cys
34480 & 34599	OPG053	IMV membrane protein L1R	Formation of intracellular mature virions (IMV) and plays a role in virion morphogenesis	Pro and Glu	Ser and Gly	Ser and Gly	Ser and Gly	Ser and Gly
38683	OPG056	EEV maturation protein	Critical for the long-range spread of the virus within host	Glu	Lys	Lys	Lys	Lys
39160	OPG057	Palmytilated EEV membrane protein	Required for IEV formation	Glu	Lys	Lys	Lys	Lys
42204	OPG062	DNA-binding phosphoprotein 1		Ile	Met	Met	Met	Met
42924	OPG063	Poly(A) polymerase catalytic subunit-3	Creates the 3′-poly(A) tail of mRNA's	Ile	Met	Met	Met	Met
50367	OPG069	Myristoylated protein E7	Reprogramming the cellular environment to be conducive to viral replication	Asn	Asp	Asp	Asp	Asp
54138	OPG071	DNA polymerase-2		Leu	Phe	Phe	Phe	Phe
54656	OPG072	Sulfhydryl oxidase	Protein disulfide bond-forming enzyme	Asp	Asn	Asn	Asn	Asn
55596, 55600, 55603 & 55606	OPG074	morphogenesis protein		Thr, Pro, Ser and Lys	Ile, Leu, Phe and Asn	Ile, Leu, Phe and Asn	Ile, Leu, Phe and Asn	Ile, Leu, Phe and Asn
64052	OPG083	Viral core cysteine proteinase	Similar to DNA topoisomerase II; Virion core cysteine protease	Phe	Ser	Ser	Ser	Ser
73087 & 73260	OPG093	Late transcription factor VLTF-1	Late gene transcription factor	Ser and Asp	Leu and Asn	Leu and Asn	Leu and Asn	Leu and Asn
74226	OPG094	Myristylated protein	Entry/fusion complex component	Met	Ile	Ile	Ile	Ile
77404	OPG098	Nucleic acid binding protein VP8/L4R	Virion core protein vp8; stimulation of I8R helicase activity & ss/dsDNA binding protein (VP8)	Glu	Lys	Lys	Lys	Lys
79427	OPG102	Cap-specific mRNA	(nucleoside-O2′-)-methyltransferase	Asn	Asp	Asp	Asp	Asp
83347	OPG105	DNA-dependent RNA polymerase subunit rpo147		Ser	Leu	Leu	Leu	Leu
93408	OPG113	mRNA capping enzyme large subunit	RNA 5′ triphosphatase and RNA guanylyl transferase activities	Lys	Glu	Glu	Glu	Glu
95957	OPG116	Uracil DNA glycosylase superfamily	DNA polymerase processivity factor	Asp	Glu	Glu	Glu	Glu
121341	OPG139	IMV membrane protein A13L	Virion maturation	Ala	Thr	Thr	Thr	Thr
124052, 124151 & 124695	OPG145	DNA helicase		Asn, Glu and Arg	Asp, Lys and Gln	Asp, Lys and Gln	Asp, Lys and Gln	Asp, Lys and Gln
128719	OPG150	Intermediate transcription factor VITF-3		Ser	Leu	Leu	Leu	Leu
140257 & 140516	OPG160	ATPase A32	DNA packaging into the virion	Asn and Ala	Ser and Ser	Ser and Ser	Ser and Ser	Ser and Ser
149289	OPG174	Hydroxysteroid dehydrogenase		Leu	Ser	Ser	Ser	Ser
154039	OPG180	DNA ligase-2		Asn	Asp	Asp	Asp	Asp
161977	OPG188	Schlafen-1	Affects the host's immune response to infection	Tyr	His	His	His	His
164856	OPG190	EEV type-I membrane glycoprotein		Ser	Pro	Pro	Pro	Pro
177790	OPG205	Ankyrin repeat protein -44	Mediate protein-protein interactions	Cys	Arg	Arg	Arg	Arg
181952 & 183491	OPG210	B22R family protein	Putative membrane-associated glycoprotein	Asp and Pro	Asn and Ser	Asn and Ser	Asn and Ser	Asn and Ser

Ala: Alanine; Arg: Arginine; Asn: Asparagine; Asp: Aspartic acid; Crm-B: Secreted TNF-alpha-receptor-like protein; Cys: Cysteine; EEV: Extracellular enveloped virus; Gln: Glutamine; Glu: Glutamic acid; Gly: Glycine; His: Histadine; IEV: Intracellular enveloped form of vaccinia virus; Ile: Isoleucine; IMV: Integral membrane protein; Leu: Leucine; Lys: Lysine; Met: Methionine; Phe: Phenylalanine; Pro: Proline; Ser: Serine; Tyr: Tyrosine; Thr: Threonine; TNF: Tumor necrosis factor; TNFRs: Tumor necrosis factor receptors; VLTF-1: Vaccinia virus late transcription factor; VITF-3: Vaccinia virus Intermediate transcription factor.

## Transmission of Monkeypox

**Figure 2.**
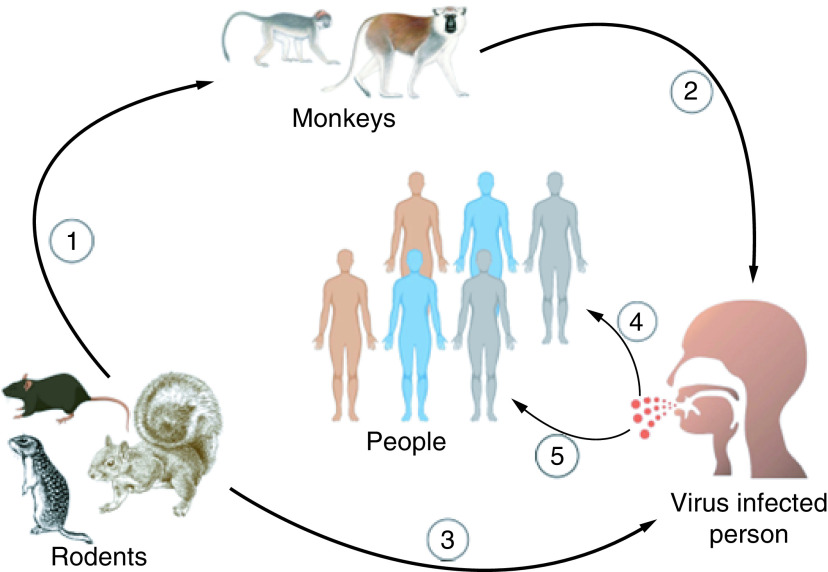
Monkeypox virus transmission. (1) From rodents to primates. (2) From primates to humans. (3) From rodents to humans. (4) From infected people to healthy people through respiratory droplets. (5) From infected persons to healthy people through direct contact.

Monkeypox virus transmission can occur either via zoonotic or humans to humans. Classically, the virus spreads mainly through prolonged exposure to respiratory droplets or close physical contact and exposure to infected body fluids [14]. Indirect transmission through direct contact with faeces and faeces carried by flies has also been suggested as possible sources of transmission among wild chimpanzees. Zoonotic transmission happens through direct contact with infected animals such as squirrels, Gambian rats, and other primates which are considered to be the natural hosts for the monkeypox infection (Figure 2) [31]. Human-to-human spread of the monkeypox virus can occur, through the respiratory droplets of infected patients as well as via physical direct contact of infected cases [32]. Direct contact can occur with the infected patient's body fluids or items and inanimate objects infected with the virus [33]. Rarely, congenital monkeypox is due to viral transmission through the placenta from mother to fetus [34]. Furthermore, recently, a new pattern of spread among sexual networks, specifically among men who have sex with men (MSM), has underscored sexual contact as a main spreading route for the virus among other potential sources [35]. the close contact with patients or with a diseased animal, immunocompromised patients may similarly be in risk of developing monkeypox [36]. Therefore, recognizing diseased cases and isolating the possibly infected persons for three weeks, is required to control the viral carry-on and spread to uninfected cases and contain the outbreak [37].

## Clinical presentation

**Figure 3.**
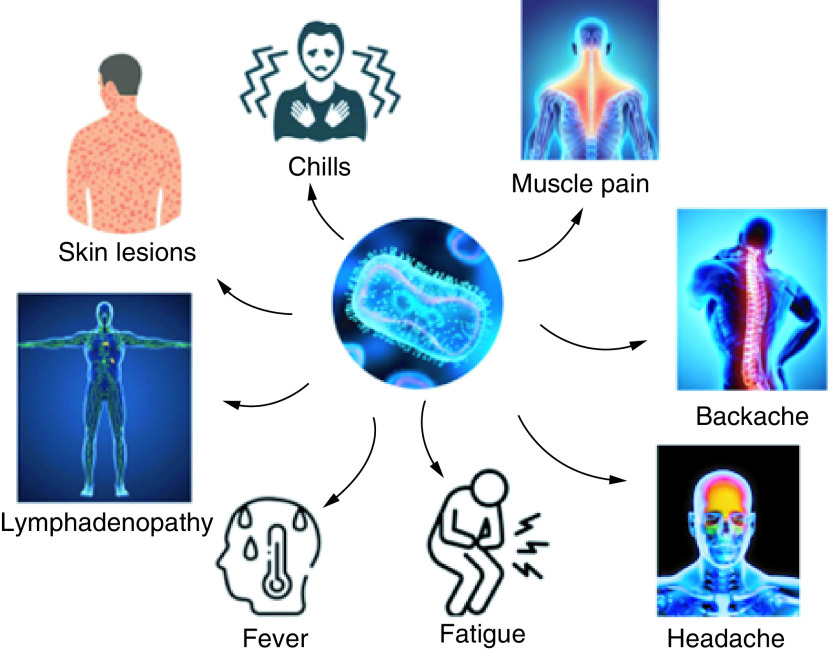
Signs and symptoms of monkeypox virus infection.

The clinical presentation of the monkeypox virus infection is similar to a milder form of smallpox however, lymphadenopathy is a characteristic clinical sign of monkeypox not present in smallpox [38]. Figure 3 shows the major clinical manifestation of the viral infection which are usually started with fever, chills, headache, muscle pains, back pain, and fatigue and then followed by the development of a rash on the face, which later disseminated to other body parts. Human monkeypox is characterized by a 5 to 21 days incubation period and three distinct phases namely, incubation, prodrome, and rash-like smallpox but with milder clinical consequences [39]. The prodromal phase is distinguished by headache, discomfort, back pain, general fatigue, sore throat, cough, and low-grade fever [40]. The rashes begin within 24 to 72 h of symptom onset [41] which appears regularly on the face and then generalizes to the palms of the hands and soles [42] and sometimes extended to other organs such as the genitals, mouth, and eyes [42]. The rash is characterized by distinctive development where it starts with a macular rash and then passes to papular rash, vesicular, and pustular lesions prior to forming a skin crust which later falls off [43] where the number of respective skin lesions can range from a little to thousands based on the immune response of the infected cases [44]. The associated skin lesions comprise infectious viruses that be capable of being spread via direct physical of sexual contact [45]. Additionally, there are several secondary complications including, gastroenteritis, sepsis, pneumonia, and encephalitis [46]. The medical cascade related to the disappearance of monkeypox may include hypertrophic and hypopigmented atrophic skin lesions and muscle contractures/deformities after facial ulcer healing [47]. Moreover, the increase in cases of monkeypox during the COVID-19 pandemic confounds the global since COVID-19 may regularly present with several serious skin manifestations like erythema multiforme, maculopapular rash, vesicular and vascular lesions, with or without flexor rash [48]. Although COVID-19 is primarily responsible for respiratory symptoms, an increasing number of cutaneous manifestations have been reported. Cutaneous manifestations are reported by patients following disease recovery. A descriptive study was done with 273 patients who had cutaneous manifestations after recovering from COVID-19. Each patient provided a thorough medical history and underwent a general physical examination. Following PCR analysis, all participants were confirmed to be COVID-19 patients. Resulting in, Acral lesions were the most common, accounting for 39% of all cases [48]. Monkeypox virus and COVID-19 are very different but both viruses cause flu-like symptoms initially. Monkeypox typically begins with a flu-like illness followed by swelling of the lymph nodes and rash on the face and body. The monkeypox virus is a relatively large DNA virus than the RNA virus. The sudden mutation of the monkeypox virus in human transmission is unlikely as DNA viruses are better at detecting and repairing mutations. COVID-19 is an RNA virus so its variants are continuously emerging and have eluded immunity from vaccination and prior infection [49]. But in the case of monkeypox, the immunity of the people from vaccination and prior infection is yet to be determined. Since atypical monkeypox and COVID-19 may disclose a comparable medical disease manifestation during the current outbreak period, COVID-19 may occasionally present with skin manifestations, such as an erythematous maculopapular rash, erythema multiforme, vesicular rash, vascular livedo reticularis, figurate erythema, or a flexural rash [48]. Because COVID-19 and atypical monkeypox in the current outbreak may share a similar clinical picture, clinicians should be vigilant about these two diseases during this time. That is why additional investigation and analysis are necessary to establish the relationship between the monkeypox COVID-19 pandemic.

## The Monkeypox virus diagnosis

The diagnostic tests are critical in confirming monkeypox infection and must be combined with medical and epidemiological data. The identification of infection caused by the monkeypox virus depends on the patient history, clinical features including signs and symptoms as well as the laboratory analysis [50]. Polymerase chain reaction (PCR) test is the gold standard of diagnosis and must be done first test on samples obtained from skin lesions, nasopharyngeal/ oropharyngeal swab, blood, and urine therefore, should be used first to confirm the suspected case of monkeypox [51]. Other tests should be done, including viral culture in which the virus is cultivated and separated from the clinical sample of the suspected cases. Further confirmation can also be established by using the electron microscope morphologically identify the viruses from clinical samples which could be tissue biopsy or vesicular liquid [52]. Also, immunochemical analysis using specific orthopoxvirus antigens and their corresponding antibodies [53]. Besides, anti-orthopoxvirus IgG and IgM serology analyses on a blood sample to determine recent or past exposure [54]. Exudate or crust from the skin lesion is gathered to isolate viral nucleic acids for diagnostic purposes by using genome-specific real-time polymerase chain reaction (RT-PCR) testing, which is the recommended test for studying the monkeypox virus during acute infection [38]. Also, western blot analysis can be conducted for further diagnostic purposes to detect specific viral proteins, particularly during acute infection with monkeypox [55].

## Prevention

Monkeypox can be spread to humans through physical contact with infected animals. Therefore, avoiding contact with infected or dead animals is of particular value to prevent acquiring the virus and avoid the development of the disease. Foods containing animal meat or parts of animal meat should be thoroughly cooked prior to consumption is also important to avoid infection with monkeypox, particularly in the endemic areas [56]. Some measures for prevention can be applied to avoid the infection of the monkeypox virus. This contains; isolating infected cases in a low-pressure room to reduce the spread of the virus from person to person, avoiding contact with any infected objects. Since, physical contact is the main route of transmission between humans, therefore, avoiding and limiting it with suspected or confirmed cases will be or particular importance in containing the viral spread and infection [57]. Front-line workers looking after monkeypox-infected individuals and others who are at high risk should wear suitable personal protective equipment and wearing N-95 mask that is suited for avoiding air-borne as well as droplet infectious [58]. In addition, wearing, entire body-covered water-resistance gowns and double-layered gloves as well as using hand hygiene with soap and water or an alcohol-based hand sanitizer is important to avoid skin-to-skin contact [59]. Effective cleaning and disinfection of possibly infected items and proper disposal and proper decontamination of contaminated waste are necessary with regard to the possible transmission of carriers of infection.

## Vaccination

Several studies have observed that vaccination against smallpox effectively prevents other *Orthopoxvirus* infections, including monkeypox, and can prevent the onset of the disease or reduce the severe clinical consequences and complications of the disease [60]. Interestingly, the commercially-valuable vaccines for smallpox are predicted to offer some immunity against monkeypox infection because of their genetic similarities. The smallpox vaccination had shown cross-immunity and therefore protection from acquiring the infection by up to 85% and lessened the sternness of monkeypox illness and the associated manifestations [61]. New-generation smallpox live-attenuated vaccines, including ACAM2000, Modified Vaccinia Ankara, and LC16m8 not only experienced an enhanced safety profile compared with first and second-generation but also induce satisfactory antibody response, particularly in the sever complicated cases such as the immunocompromised patients [62]. On the other hand, the protection from past smallpox immunization will not of help during the current epidemics since it was restricted to those who were administered the vaccine by or before the 1980s and, over the last four decades, there is every opportunity of additional decrescent of the defensive effect of such old vaccinations [63]. To control the present epidemic of the monkeypox virus, health sectors have appointed rules to give smallpox vaccines for healthcare practitioners handling infected patients in different nations [63]. In 2019, the Modified Vaccinia Ankara-Bavarian Nordic strain (MVA-BN) which is considered a third-generation smallpox vaccine, was permitted for use against monkeypox which is produced based on a strain of vaccinia virus [64]. On 24 May 2022, the US CDC decided to commute some of their MVA-BN (known as JYNNEOS vaccine), to persons at high risk of developing this viral disease but not to the general public [65]. Moreover, non-replicating (MVN-BN) and minimally replicating (LC16) vaccines are recommended for children, and pregnant and breastfeeding women [65]. The LC16 vaccine is the only authorized vaccine for infants and children. On the other hand, the MVA-BN vaccine is approved for adults [66]. The guidelines propose that the safety profiles of the accessible vaccines should be taken into consideration by the medical experts to evaluate the benefit-risk ratio before taking the decision if vaccine demonstration [67]. Public health measures and vaccination for high-risk groups were recommended by the WHO to contain the outbreak. As of 16 January 2022, more than 83,000 monkeypox (formerly known as monkeypox) cases have been confirmed worldwide in 102 non-endemic countries [68]. A pre-exposure vaccination campaign to prevent the spread of the monkeypox virus was initiated in Italy in August 2022. The study explores the possible factors affecting the trend of monkeypox cases in an Italian region (Lazio) with a rapid roll-out of the vaccination campaign. The analysis of surveillance data showed a significant decreasing trend in the number of monkeypox cases starting from the second week after vaccination [69]. The reported trend in monkeypox cases is likely to result from a combination of multiple social and public health factors combined with a vaccination campaign.

## Management

Monkeypox illness generally incites slight symptoms, self-limiting, and for the most part patients recuperate without treatment. According to the CDC guidelines, there is at present no particular therapy for monkeypox virus infections. In spite of this, antiviral drugs endorsed to cure smallpox may be utilized to cure monkeypox [70]. Tecovirimat (ST-246) is an antiviral drug employed for the therapy of smallpox disease, and is authorized by the Food and Drug Administration (FDA) to be used to remedy monkeypox at the time of an outbreak [71]. Tecovirimat is an oral and intravenous injection that targets a highly conserved Orthopoxvirus envelope protein (F13L) and blocks virion release in the treatment of monkeypox [72]. Another two antiviral agents namely, cidofovir and brincidofovir, also evolved to cure smallpox and act by preventing the viral DNA polymerase that results in the inhibition of poxvirus DNA replication, have shown efficacy in animal observations [73]. Furthermore, brincidofovir, a cidofovir analog that is also accessible orally, was recently approved for the treatment of human cytomegalovirus infection [74]. Based on the information gained upon drug use in a controlled number of human cases of monkeypox suggested that tecovirimate is more effective, while brincidofovir has low efficacy in the treatment of monkeypox [75].

Although Vaccinia Immune Globulin Intravenous (VIGIV) is applied to treat serious side effects of resulted from vaccinia vaccination, it is recommended for the therapy of monkeypox during recent epidemics [76]. VIGIV is crucial in post-exposure prophylaxis and in the reduction of the disease severity, however, additional clinical studies are still required to investigate and prove its efficacy and safety. Finally, the use of these medications in endemic areas in the management of certain monkeypox infections can be respected, and the doctors are permitted to pursue these choices relying upon the situation and severity of the cases [77].

## Conclusion

The monkeypox disease rises in non-endemic locations indicating that the pathogens are not limited to geographical borders. The current monkeypox outbreak besides the COVID-19 pandemic has become another danger and increased fear among the population worldwide. Therefore, a global health framework should develop compelling strategies as well as strict protective and control measures should be undertaken to alleviate the spread of viral infection. The monkeypox uprising has been a focal point of regard for researchers, clinicians, epidemiologists, and policy pioneers. Nonetheless, priority ought to be given to control efforts that should depend on upgraded case finding, contact tracing, isolation, good hygiene practices, and immunization. Although the viral members of genus *Orthopoxvirus* are characterized by their genomic stability, however, based on the complete genome sequences of the currently circulating monkeypox strain there is evidence of 34 nonsynonymous missense SNPs of the monkeypox strain isolated in 2022 as compared with the USA, Spain, Japan and South Africa strains. Whether these SNPs are associated with the magnitude of the viral pathogenicity, the spread of infection as well as in protection efficiency of the commercially available vaccines still has to be clinically investigated.

## Future perspective

Several necessary protective measures and responses to potential occurrences in light of the recent monkeypox pandemic should be strictly implemented to contain this newly emerged vial pandemic. Although this virus is characterized by a genetically stable genomic DNA however, there is evidence of newly emerged mutation resulting in nonsynonymous missense single nucleotide polymorphism (SNP) in 34 ORFs (19.1%) out of the 178 ORFs of the complete genome in the genomic DNA of the monkeypox strain that was recently isolated in Egypt. the complete genome of the USA, Spain, Japan, and South Africa strains showed no SNPs when they were compared with each other. The SNPs detected in each ORF were either one, two, three, or four missense SNPs, with a percentage of, 79.4%, 11.8%, 8.8%, and 2.9%, respectively. Whether these SNPs will be associated with the pathogenicity and spread of infection as well as in the protection efficiency of the commercially available vaccines still has to be investigated and biochemically identified in the future.

**Executive summary**

- Viruses are still emerging and responsible for many new cases of medically significant recurrent infections and widespread sets of human and animal infectious diseases worldwide.
- The 2022 monkeypox outbreak has created a new global health threat and pandemic. Monkeypox virus is a descendant of the genus *Orthopoxvirus*, producing a febrile rash disease.
- Monkeypox is zoonotic transmitted and transmitted from human to human in several ways.

**Genomic mutation of the circulating viral strains**

- Comparative genomic analysis of the monkeypox strains that have been recently isolated in the USA, Spain, Japan, and South Africa showed no detected nonsynonymous SNPs when they were compared with each other. However, the SNPs were determined in 34 open reading frames (ORFs) of the complete genome of the Egyptian strain out of the 178 ORFs (19.1%) of the complete genome when compared with the recently isolated USA strain in 2022.
- The SNPs detected in each ORF were either one, two, three, or four missense SNPs, with a percentage of, 79.4%, 11.8%, 8.8%, and 2.9%, respectively.
- Whether these SNPs are associated with the pathogenicity, spread of infection as well as in protection efficiency of the commercially available vaccines still has to be investigated and biochemically identified.

**Diagnosis of monkeypox**

- Diagnostic tests are critical in confirming monkeypox infection and must be correlated with clinical and epidemiological information.
- Both conventional PCR and RT-PCR tests can be used to confirm the suspected case of monkeypox and to evaluate the viral burden in blood, respectively.
- Morphological identification of the poxviruses can be done using the electron microscope from a tissue biopsy or vesicular exudate.
- Immunochemical examinations are performed for the existence of specific antigens of the Orthopoxvirus.
- The monkeypox immunoglobulins IgG and IgM serology tests on a blood sample to identify recent or past exposure.
- Western blot analysis of certain viral proteins can be used to confirm the diagnosis.

**Vaccination of monkeypox**

- The smallpox vaccine is predicted to provide partial immunity against monkeypox infection due to their genetic similarities and had shown up to 85% cross-protection.
- The live attenuated vaccines including, ACAM2000, Modified Vaccinia Ankara, and LC16m8 are new-generation smallpox vaccines with improved safety profiles compared with first and second-generation vaccines and arouse satisfactory antibody production.
- To control the current outbreak of the monkeypox virus, health sectors have appointed policies to dispense smallpox vaccines for healthcare practitioners.
- Modified vaccinia Ankara-Bavarian Nordic strain (MVA-BN), a third-generation smallpox vaccine was permitted in 2019 against monkeypox.
- On May 24, 2022, the US CDC decided to commute some of their MVA-BN, for persons at high risk of coming in contact with monkeypox virus.
- A non-replicating (MVN-BN) and minimally replicating (LC16) vaccines are recommended for children, and pregnant and breastfeeding women.

**Management of monkeypox**

- There is currently no particular treatment for monkeypox virus infections however CDC advised the use of anti-smallpox drugs to be used to treat monkeypox disease.
- Tecovirimat (ST-246) is available orally (200 mg capsule) and was authorized by the Food and drug administration (FDA) to cure human smallpox and monkeypox in adults and pediatric patients.
- Both cidofovir and brincidofovir act by inhibiting the viral DNA polymerase and are also evolved to treat smallpox and monkeypox.
- Serious side effects due to vaccinia vaccination, such as eczema vaccinium, severe generalized vaccinia, and vaccinia virus-induced infections can be managed by using Vaccinia Immune Globulin Intravenous (VIGIV).
